# Equivalence study of the resin-dentine interface of internal tunnel restorations when using an enamel infiltrant resin with ethanol-wet dentine bonding

**DOI:** 10.1038/s41598-024-63289-0

**Published:** 2024-05-30

**Authors:** Andrej M. Kielbassa, Sabrina Summer, Wilhelm Frank, Edward Lynch, Julia-Susanne Batzer

**Affiliations:** 1 Centre for Operative Dentistry, Periodontology, and Endodontology, Department of Dentistry, Faculty of Medicine and Dentistry, Danube Private University (DPU), Steiner Landstraße 124, 3500 Krems an der Donau, Austria; 2 Department for Biomedical Research, Centre of Experimental Medicine, University for Continuing Education Krems, Krems an der Donau, Austria; 3 Centre for Health Sciences, Department of Medicine, Faculty of Medicine and Dentistry, Danube Private University (DPU), Krems an der Donau, Austria; 4 Leicester School of Pharmacy, De Montfort University, Leicester, UK

**Keywords:** Confocal laser scanning microscopy, Dental adhesives, Dentine bonding, Ethanol-wet bonding, Hybrid layer, Icon, Resin infiltration, Scanning electron microscopy, Scotchbond multi-purpose, Split-tooth design, Syntac, Biotechnology, Health care, Medical research, Materials science

## Abstract

This preregistered ex vivo investigation examined the dentinal hybrid layer formation of a resinous infiltrant (Icon), with reference to both thickness (HLT) and homogeneity when combined with modified tunnel preparation (occlusal cavity only) and internal/external caries infiltration. The adhesives Syntac and Scotchbond MP were used as controls (Groups 1 and 3) or in combination with Icon (Groups 2 and 4). A split-tooth design using healthy third molars from 20 donors resulted in 20 prepared dentine cavities per experimental group. The cavity surfaces (n = 80) were etched (37% H_3_PO_4_), rinsed, and air-dried. Rewetting with ethanol was followed by application of the respective primers. After labeling with fluorescent dyes, either Syntac Adhesive/Heliobond or Scotchbond MP Adhesive was used alone or supplemented with Icon. HLT, as evaluated by scanning electron microscopy, did not significantly differ (P > 0.05), and confocal laser scanning microscopy revealed homogeneously mixed/polymerized resin-dentine interdiffusion zones in all groups. Icon can be successfully integrated into an ethanol-wet dentine bonding strategy, and will result in compact and homogeneous hybrid layers of comparable thickness considered equivalent to the non-Icon controls, thus allowing for preservation of the tooth’s marginal ridge and interdental space in the case of internal/external infiltration of proximal caries.

## Introduction

Previously, both the development and the introduction of the resin infiltration technique^1–3^ have resulted in a noninvasive treatment regimen for (proximal) enamel caries aimed at closing the gap between preventive and restorative dentistry, thus obviating any sacrifice of dental tissues and improving oral health^4^. When the low-viscosity resin used in this treatment regimen has been applied to deproteinized enamel lesions^5^, major parts of the porous volume of these lesions may be occluded, thereby leading to the construction of a three-dimensional network^1^ consisting of the infiltrant resin enwrapping the demineralized enamel remnants, rehardening and stabilizing the lesion^6^, and paralyzing any progress in the latter. Meanwhile, several independent meta-analyses of acceptable quality^7–12^ (including an umbrella review^13^) have consistently revealed that resin infiltration of enamel lesions will be effective at reducing any further clinical increase in white-spot lesions in both dentitions, as well as providing prompt and acceptable aesthetic outcomes^4^. However, this refers to lesions restricted to enamel only, and it should be highlighted that lesions extending beyond the enamel–dentine junction have never been assessed with the initial developments^1,2^.

Notably, some studies have revealed a poor outcome of the infiltration technique when used for treating proximal lesions extending into the outer third of the dentine, with the therapeutic efficacy of resin infiltration considered not to be significantly different from that of the controls^10^. Consequently, radiographically visible lesions extending to the outer dentine do constitute either a contraindication for the resin infiltration technique or a need for a modified treatment regimen. The latter has been introduced recently^14,15^ and combines the modified (internal) tunnel technique with both internal and external resin infiltration of the carious enamel lesion. By means of an occlusal approach, dentine caries can be removed, and enamel caries can be infiltrated both from inside the cavity and from the proximal site, thus obstructing porous enamel lesion areas by a doubled infiltration through capillary forces. This leads to a stabilization of the weakened proximal enamel^14,15^ and should result in increased clinical success rates. When reflecting on the final restoration of the occlusal access cavity, an adhesively bonded resin restoration would seem recommendable.

However, to date, no information regarding the morphology of the bonding interface between dentine, dentine bonding agent, caries infiltrant, and resinous restoration has been obtained from the literature. With the various components of the adhesives in mind, compatibility would seem conceivable, and, hence, the present ex vivo study aimed to analyze various aspects of the resin-dentine interface after the use of a resin infiltrant (Icon Infiltrant; DMG, Hamburg, Germany) in combination with two commercially available etch-and-rinse adhesive systems (Syntac [Ivoclar Vivadent, Schaan, Liechtenstein] and Adper Scotchbond Multi-Purpose [3M/ESPE, St. Paul, MN, USA]). The primary aim was to study hybrid layer formation (when combining the modified tunnel preparation with internal/external resin infiltration of proximal caries lesions), and our null hypothesis (H_0_) stated that the difference between the means of neither the hybrid layer thickness nor the homogeneity of the latter would be outside the equivalence interval when evaluated by means of scanning electron microscopy (SEM) and confocal laser scanning microscopy (CLSM), respectively. The working hypothesis (H_A_) claimed the opposite and stated that, with respect to the quality of the hybrid layer, the difference between the means would be beyond the bounds of the equivalence interval (meaning that the effect size would indicate less than a small but negligible difference). The secondary aims pertained to aspects concerning both adhesive layer thickness and resin tag formation.

## Methods

### Ethical approval and trial registration

With the current report, we adhered to the modified CONSORT guidelines for reporting preclinical studies on dental materials^16,17^. This study protocol was approved by the Institutional Ethical Review Board of Danube Private University (DPU) in Krems, Austria (vote number: DPU-EK/025; date of approval: 23 March 2023). Prior to the start of the study, the protocol of the current setup was registered at https://www.protocols.io/blind/E5754DAFD67D11EEB9800A58A9FEAC02; the date of registration was 17 April 2023. After extraction, the teeth were anonymized, and we obtained unrestricted permission for the use of this kind of human body material for both research and publication^18^.

### Sample size calculation and tooth selection

In the present study, the well-accepted multibottle etch-and-rinse adhesive systems Syntac (Ivoclar Vivadent) and Adper Scotchbond Multi-Purpose (3M/ESPE; hereafter called Scotchbond MP), respectively, served as positive controls; with these conventional adhesive systems, reliable data are available for the analysis of the micromorphology of the water-wet bonded resin-dentine interface created on healthy dentine^19,20^. However, the proprietary adhesive components of these systems (Heliobond [Ivoclar Vivadent] or Scotchbond MP Adhesive [3M/ESPE]) have not yet been supplemented by the resinous caries infiltrant (Icon-Infiltrant; DMG); thus, a sample size calculation was not possible. Consequently, the estimation of the number of samples and their preparation followed the “Academy of Dental Materials Guidance” on in vitro testing of the effectiveness of dental composite bonding^21^. This guideline recommends the use of 10 teeth per group to study tensile bond strength; to ensure a reliable result with the present pilot study focusing on the resin-dentine interface, the number of samples included per group was doubled.

Twenty freshly extracted maxillary and mandibular human third molars (numbered consecutively from 1 to 20), without caries, cracks, restorations, or root canal treatment and without any other pathological changes (e.g., hypomineralization, fluorosis, and/or erosion), which had at least partially erupted into the oral cavity and showed a widely completed apical root development were obtained from 20 patients (mean [SD] age 21.1 [2.4] years; age range 16–25 years). The latter provided written informed consent following verbal information. In the case of donors younger than the legal age, a parent or legal guardian signed the consent form. The patients’ teeth were subsequently included in the present research project.

**Figure 1:**
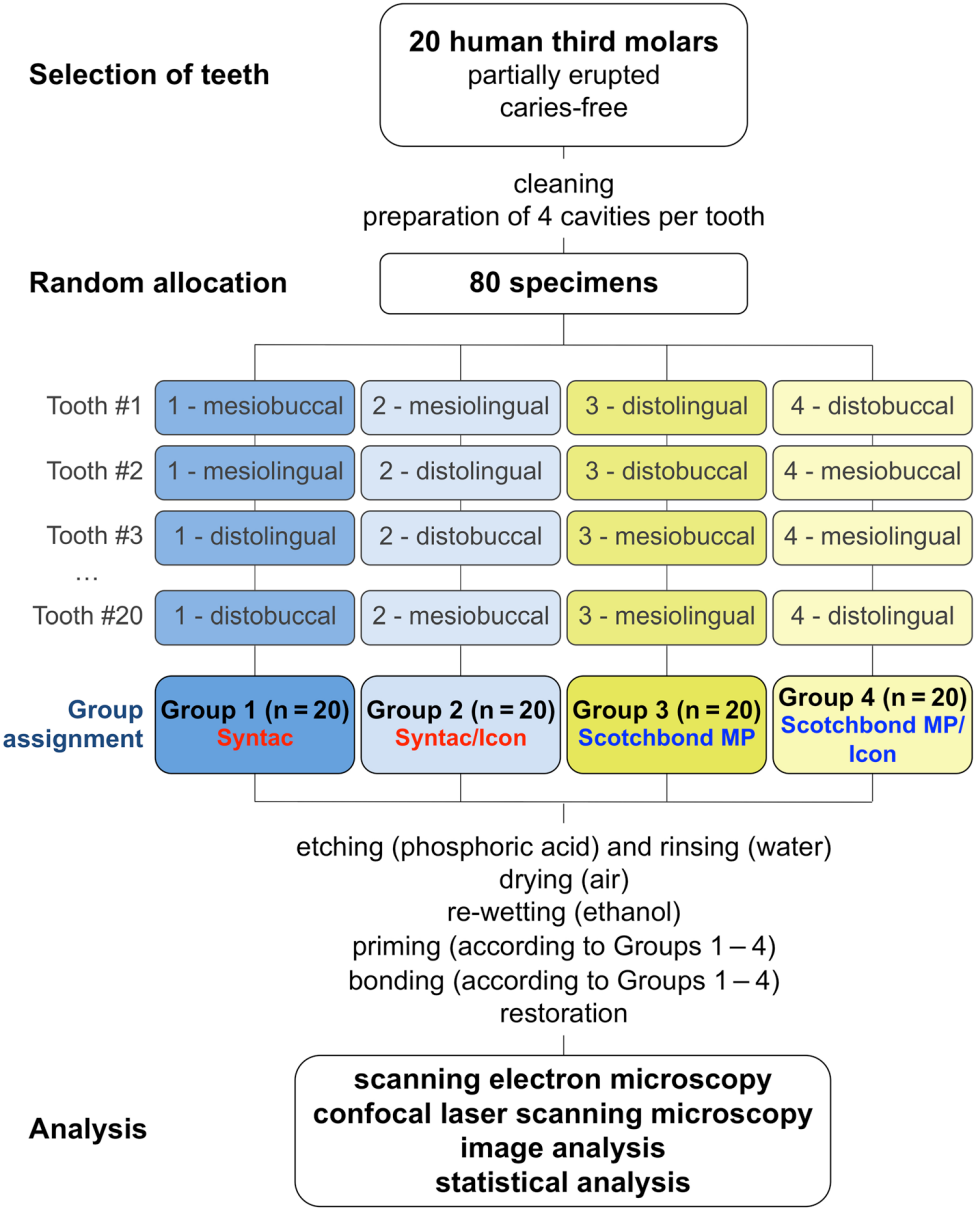
Flowchart presenting the group assignment/randomization and experimental setup.

Following careful cleaning of all residual adherent external natural deposits (e.g., calculus, biofilm, pellicle, and/or soft tissues) with a tap water-cooled dental ultrasonic scaler (Teneo; Dentsply Sirona, Bensheim, Germany), the teeth were stored in physiological saline (0.9% NaCl solution at pH 7; Gottlöber Pharmacy, Sandersdorf, Germany) at room temperature in hermetically sealed boxes (Labordose; Bürkle, Bad Bellingen, Germany) until use. Following a previously published guideline^21^, the time between extraction and experimental procedures was kept as short as possible (less than 2 months). For the implementation of the split-tooth design used in the present study, each of the four tooth quarters (mesiobuccal, mesiolingual, distolingual, and distobuccal) of the 20 teeth (n = 80 specimens) was assigned to one of the four bonding strategies to be tested, thus ensuring a uniform distribution of the respective tooth quarters. The sequence presented in Fig. 1 was concealed prior to any assignment of interventions (as described in detail below). Consequently, the tooth (and the donor) was the statistical unit, and each tooth allocation was assigned at random, thus following the prespecified order (Fig. 1). All tooth preparations and restorative procedures were conducted using magnifying glasses (opt-on, 2.7 × TTL, 350 mm; orangedental, Biberach, Germany).

### Preparation of the teeth

All cutting and preparation operations were carried out under continuous cooling using tap water. First, the crowns of each tooth (n = 20) were separated from the roots 2 mm below the cement-enamel junction and perpendicular to the long axis of the tooth using a thin double-sided, diamond-coated cutoff wheel (#H355SF220, Superdiaflex SF, extrafine, Ø 22.0 mm, thickness 0.22 mm; Horico Dental, Berlin, Germany) fixed in a straight handpiece (40,000 rpm, T1 Line H 40 L, transmission ratio [TR] 1:1; Dentsply Sirona), thus exposing the pulp chamber. The pulp tissue was removed with a dental scaler (SM239E2—#23 Gr #9; Hu-Friedy, Chicago, IL, USA).

Subsequently, with each tooth, an occlusal orientation groove of 1 mm depth was prepared in the region of the deepest pit of the central fissure by using a cylindrical, diamond-coated bur (#DM10.314.009, PrepMarker, 107 µm, axial cutting depth of 1 mm; Komet Austria, Salzburg, Austria), driven by a red contra-angle handpiece (160,000 rpm, T1 Line C 200 L, TR 1:5; Dentsply Sirona). An occlusal Class I cavity, central to the marginal ridges, with a maximum dentine depth of 1 mm (proceeding from the deepest pit of the fissure system) and with a box-shaped extension to the maximum extent of the dental crown, was prepared with medium diamond-coated drills (#959KRD.314.018 and #845KRD.314.025, 107 µm; Komet Austria; 160,000 rpm, T1 Line C 200 L [red contra-angle handpiece], 1:5; Dentsply Sirona).

The exposed dentine surface at the cavity floor was checked carefully to ensure that the pulp chamber had not been opened during preparation. Then, each dental crown was sectioned both in mesial-distal and in lingual-buccal directions (perpendicular to the occlusal tooth surface), thus dividing the Class I cavity and the underlying tooth substance into four more or less equally sized parts using a double-sided, diamond-coated cutoff wheel (#H355SF220, Superdiaflex SF, extrafine, Ø 22.0 mm, thickness: 0.22 mm; Horico Dental; 40,000 rpm, T1 Line H 40 L [straight handpiece], TR 1:1; Dentsply Sirona). The diamond-coated burs and the cutoff wheels were replaced after every fifth tooth.

### Bonding procedures

Two quarters of each cavity were assigned to Group 1 or Group 3 (control groups treated with either Syntac or Scotchbond MP), respectively, and were treated by means of a modified adhesive protocol (as described below). The remaining quarters of each tooth were assigned to Group 2 or Group 4, respectively; with these experimental groups, a modified bonding process using infiltration resin (Icon) was introduced (Fig. 1). The ingredients of the two conventional adhesive systems as well as those of the resinous caries infiltrant are listed in Table 1 (information according to the respective manufacturers), while Table 2 provides a brief overview of the application procedures of the four bonding strategies used with the present setup. Before starting the adhesive restoration of each tooth quarter, the dissected dentine surfaces were sealed (using a die/color spacer stump varnish, 7 µm; Yeti Dental, Engen, Germany) to avoid any daubing and to allow the penetration of the bonding agents into the dentine structures via the cavity surface only.

**Table 1:** Composition of the adhesive systems and the caries infiltrant resin used in the present study (information provided by the manufacturers with the respective safety data sheets).

Product (manufacturer)	Components [specifications refer to percentages by weight]	Batch/lot number
Syntac (multibottle HEMA-free etch-and-rinse adhesive; Ivoclar Vivadent, Schaan, Liechtenstein)		
Primer	Triethylene glycol dimethacrylate (TEGDMA) [10 to < 20%] Poly(ethylene glycol) dimethacrylate (PEGDMA) [3 to < 7%] Maleic acid [3 to 5%] Acetone [30 to < 50%] Water	Z03Y4P
Adhesive	Poly(ethylene glycol) dimethacrylate (PEGDMA) [25 to 50%] Glutaraldehyde [3 to < 10%] Water	Z03R90
Heliobond	Bisphenol-A diglycidyl methacrylate (Bis-GMA) [50 to 100%] Triethylene glycol dimethacrylate (TEGDMA) [25 to 50%] Stabilizers Camphorquinone	Z03SSH
Scotchbond MP (two-bottle etch-and-rinse adhesive containing HEMA; 3M/ESPE, St. Paul, MN, USA)		
Primer	2-Hydroxyethyl methacrylate (HEMA) [35 to 45%], Copolymer of itaconic and acrylic acids [10 to 20%], Water	NE86870
Adhesive	Bisphenol-A diglycidyl methacrylate (Bis-GMA) [60 to 70%], 2-hydroxyethyl methacrylate (HEMA) [30 to 40%], Ethyl 4-dimethyl aminobenzoate (EDMAB) [< 0.5%], Triphenylantimony [< 0.5%], Triphenylphosphine [< 0.2%], Hydroquinone [< 0.5%]	NE86404
Icon-Infiltrant (caries-infiltrating resin; DMG, Hamburg, Germany)		
	Triethylene glycol dimethacrylate-based resin matrix (TEGDMA) [≈ 78%], Trimethylolpropantriacrylat [20%], (2-ethyl-hexyl)-p-dimethylaminobenzoat [< 1%], 2,6-di-tert-butyl-4-methylphenol [< 1%], Camphorquinone (< 1%), additives^132^	247113 271920

**Table 2:** Adhesive working steps for the various experimental groups.

	Dentine conditioning	Priming/wetting procedure	Bonding procedure
Group 1 (Syntac; n = 20)	H_3_PO_4_ gel (60 s) C_2_H_5_OH (60 s)	Syntac Primer (15 s) Syntac Adhesive (10 s)	Heliobond (3 min) Final air-drying (20 s)
Group 2 (Syntac/Icon; n = 20)	H_3_PO_4_ gel (60 s) C_2_H_5_OH (60 s)	Syntac Primer (15 s) Syntac Adhesive (10 s)	Icon Infiltrant (3 min) Heliobond (20 s) Final air-drying (20 s)
Group 3 (Scotchbond MP; n = 20)	H_3_PO_4_ gel (60 s) C_2_H_5_OH (60 s)	Scotchbond MP Primer (15 s)	Scotchbond MP Adhesive (3 min) Final air-drying (20 s)
Group 4 (Scotchbond MP/Icon; n = 20)	H_3_PO_4_ gel (60 s) C_2_H_5_OH (60 s)	Scotchbond MP Primer (15 s)	Icon Infiltrant (3 min) Scotchbond MP Adhesive (20 s) Final air-drying (20 s)

*H*_*3*_*PO*_*4*_ phosphoric acid gel, 37% (Total Etch Gel; Ivoclar Vivadent, Schaan, Liechtenstein), *C*_*2*_*H*_*5*_*OH* ethanol, < 100% (Icon Dry; DMG, Hamburg, Germany); for the manufacturers of the other used materials, see Table 1.

Prior to application, 2 drops of Syntac Adhesive (Group 1) were labeled with 10 µl of a green fluorescing agent (0.1 mmol fluorescein isothiocyanate [FITC]; Gottlöber Pharmacy), while 2 drops of Heliobond (Group 1) and Icon (Group 2) were marked with 10 µl of a red fluorescing agent (0.1 mmol rhodamine B isothiocyanate [RITC]; Gottlöber Pharmacy). For this reason, 10 µl of the respective fluorescing agent was pipetted (Pipette Research plus; and Pipette tips epT.I.P.S, 100 µl; Eppendorf, Hamburg, Germany) into the hollow of a small mixing palette (#2315, mixing palette; VOCO, Cuxhaven, Germany) and mixed with Syntac Adhesive or Heliobond (and Icon, respectively). Similarly, 2 drops of Scotchbond MP Primer (Group 3) were labeled with 10 µl of FITC (0.1 mmol), and 2 drops of Scotchbond MP Adhesive (Group 3) were mixed with 10 µl of RITC (0.1 mmol). In contrast, for Group 4, 2 drops of Scotchbond MP Primer were mixed with 10 µl of FITC (0.1 mmol), and 2 drops of Icon were labeled with 10 µl of RITC (0.1 mmol). Before mixing in the pigment, the ethanol (96%) content was completely evaporated, and only the dry pigment was still present.

With reference to the split-tooth design (n = 20 teeth, with four groups per tooth; n = 20 specimens per experimental group), each of the four dentine cavity quarters was adhesively restored after cavity preparation. After preparation and prior to restoration, the cavities were carefully rinsed and dried using a dental air/water sprayer (Sprayvit, Teneo; Dentsply Sirona; 30 s each). The dentine surfaces of the cavities (all 80 samples) were first etched with phosphoric acid gel (Total Etch Gel, 37%; Ivoclar Vivadent) for 60 s. The etchant was then washed off with water spray (Sprayvit) for 30 s, and the cavity was again thoroughly dried with compressed air (Sprayvit) for 15 s. Subsequently, an ethanol-wet bonding technique was used with both the control groups and the experimental groups. For this purpose, all the cavities were additionally treated with pure ethanol (< 100%, Icon Dry; DMG, Hamburg, Germany), which was actively applied using a microbrush (Microbrush Plus, superfine white, Ø 1.0 mm; Microbrush International, Grafton, WI, USA) for 1 min. Excess ethanol was gently evaporated by means of a gentle stream of air (Sprayvit) for 10 s, and it was ensured that all the dentine surfaces were kept slightly wet and had moist gloss.

Subsequently, by using a microbrush, Syntac Primer (Tables 1 and 2) was actively rubbed onto the ethanol-wet dentinal surfaces of the specimens in Group 1 for 15 s, and excess Syntac Primer was dispersed and slightly dried for 5 s with a dental air/water syringe (Sprayvit), thus ensuring a slight excess of the primer. This was followed by the application of FITC-labeled Syntac Adhesive (Tables 1 and 2) via a microbrush (Ø 1.0 mm). Ten seconds after mixing well and massaging in, the Syntac Adhesive was carefully blown with a compressed air stream (Sprayvit; 5 s) until a shiny surface could still be determined. In the third step, RITC-labeled Heliobond (Tables 1 and 2) was applied to the dentine surfaces and massaged in for 3 min using a microbrush (Ø 1.0 mm) to allow the bonding agent to extensively penetrate the demineralized collagen. Any excess material was carefully dabbed off with a new microbrush, which was wiped over the cavity floor without pressure, and air-blasting to evaporate any solvents from the adhesives was performed for 20 s (Sprayvit)^22^; light-curing (> 1250 mW/cm^2^, Mini LED Curing Light; Satelec/Acteon Group, Mérignac, France) was performed for 40 s^23,24^, while a 3 mm working distance between the light source and the cavity floor was ensured.

The etched, rinsed, and dried (but ethanol-wet) dentine cavities of Group 2 were conditioned with the nonlabeled Syntac Primer, followed by the application of the FITC-labeled Syntac Adhesive as described above. In contrast to Group 1, the RITC-labeled caries infiltrant resin (Icon; see Tables 1 and 2) was used. By ensuring a penetration time of 3 min, the infiltration resin was massaged in (finally followed by active application of Heliobond for 20 s). Subsequently, air-drying was performed for 20 s (Sprayvit), and all the components were polymerized for 40 s (> 1250 mW/cm^2^, 3 mm working distance, Mini LED Curing Light; Satelec).

With the specimens in Group 3, the etch-and-rinse procedure was first performed on the dentine cavities as described for Group 1. After the dentine surface was rewet with ethanol, FITC-labeled Scotchbond MP Primer (Tables 1 and 2) was applied for 15 s with a Microbrush Plus (Ø 1.0 mm) to create a glossy, moist dentine surface, followed by an air-driven distribution for 5 s (Sprayvit). Then, RITC-labeled Scotchbond MP Adhesive (Tables 1 and 2) was actively massaged onto the dentine surface for 3 min using a microbrush (Ø 1.0 mm), air-blown to a thin layer (Sprayvit; 20 s), and light-cured at a 3 mm working distance for 40 s (> 1250 mW/cm^2^, Mini LED Curing Light; Satelec).

In Group 4, the etched, rinsed, dried, and rewetted dentine cavities were conditioned with FITC-labeled Scotchbond MP Primer as described for Group 3 (15 s). RITC-labeled Icon (Tables 1 and 2) was then applied to the ethanol-wet dentine surface of the cavities and actively massaged in for 3 min (but not light-cured). The Scotchbond MP Adhesive was then actively applied onto the dentine surface using a microbrush (Ø 1.0 mm) for 20 s, air-dried to a thin layer (Sprayvit; 20 s), and polymerized as described above (> 1250 mW/cm^2^, 3 mm working distance, Mini LED Curing Light; Satelec).

### Restoration placement

Subsequently, each cavity quarter was restored with a pure light-curing and pasty nanohybrid composite restoration (shade P-A3, G-ænial A'CHORD; GC Europe, Leuven, Belgium). To avoid air bubbles in the restorative material and at the cavity margins, the composite resin was carefully applied to both the cavity floor and the walls with the spherical end of a WHO probe (UNC15/11.5B; Hu-Friedy). The restoration was polymerized for 30 s (> 1250 mW/cm^2^, Mini LED Curing Light; Satelec) with a working distance of 3 mm between the light source and the restorative material. Until the microscopic analyses, the 80 restored tooth quarters were placed in membrane boxes (Hager & Werken, Duisburg, Germany) containing cotton balls (cotton balls, size 0; Omnident, Rodgau, Germany) soaked in 0.9% saline (Gottlöber Pharmacy) to ensure that the specimens were kept in a moist but not wet environment. Attention was given to further proceeding and microscopic examination of all the restored specimens within 3 days.

### Specimen preparation for microscopy

The four adhesively restored segments (mesiobuccal, mesiolingual, distolingual, and distobuccal) of each dental crown (n = 80) were cleaned with a small spatula (TNPFI8A—#8A GR #6S XTS; Hu-Friedy) to remove the previously applied stump varnish. Polishing of the resin-dentine composite surface was ensured with a rubber polisher (#DM5595293, AABA-Dental Universal Polisher; Kerr Dental, Kloten, Switzerland) driven by a blue contra-angle handpiece (40,000 rpm, T1 Line C 40 L, TR 1:1; Dentsply Sirona) under continuous water cooling; this procedure was performed for 60 s parallel to the surface to be examined microscopically. After polishing, the surfaces were etched with 15% hydrochloric acid (Icon Etch, 15% HCl; DMG, Hamburg, Germany) for 5 s to visualize the hybrid layer and the adhesive layer; before the microscopic examination, the acid (HCl) was gently removed using a dental air/water sprayer for 10 s (Sprayvit, Teneo; Dentsply Sirona). This was followed by microscopic analyses.

To visualize the resin tag formation in the dentinal tubules, selected samples were etched with hydrochloric acid (Icon Etch, 15% HCl; DMG) for 30 s. This was followed by rinsing with 5 ml of ethylenediaminetetraacetic acid (18% EDTA; Ultradent, Cologne, Germany) and with 5 ml of sodium hypochlorite (3% NaOCl; Hedinger, Stuttgart, Germany), both for 30 s. Possible remnants were carefully removed by in-between use of a dental air/water sprayer (Sprayvit, Teneo; Dentsply Sirona) for 10 s after etching and rinsing with EDTA and NaOCl.

### Scanning electron microscopy (SEM) and image analysis

With each adhesively restored cavity quarter (n = 80), the resin-dentine interface morphology in the central part of the mesiodistal interface directly adjacent to the two cut surfaces was examined by means of a scanning electron microscope (SEM, FlexSem 1000 II; Hitachi High Technologies, Tokyo, Japan) working in low-vacuum mode at ≥ 400× magnification at 20 kV. Prior to imaging, the samples were coated with gold at 30 mA for 15 s using a Q150T ES Plus sample sputter coater (Quorum Technologies, Laughton, UK). To ensure that the same areas were evaluated with the different microscopic techniques, a small indentation was prepared in each sample; for this purpose, a diamond cutoff wheel was used to vertically scratch 2 mm from the endpoint of the specimen. The images were stored on a suitable storage medium (WD Elements Portable external hard disc; Western Digital, San José, CA, USA) and analyzed using an open-source image processing and analysis program (ImageJ 1.35 S software, National Institutes of Health [NIH]; Bethesda, MD, USA). For accurate SEM measurements of the thickness of the hybrid and the adhesive layers, measurements were taken at 3 different areas per image (those were located 2 mm to the right and left of the image border and in the center of the image, respectively). If these measuring points coincided exactly with a resin tag, the measurement point was moved to the right until it could be placed between two resin tags. The hybrid layers and adhesive layers were measured at 400× magnification with ImageJ software. For the statistical analysis, all the data were collected in an Excel file (Microsoft Excel; Microsoft, Redmond, WA, USA; available at: https://office.microsoft.com/excel). The examination of open dentine tubules and resin tags was limited by the presence of the tubules or whether tag formation had occurred; resin tag formation was investigated with selected specimens using the SEM at 650× magnification.

### Confocal laser scanning microscopy (CLSM) and image analysis

In addition, each sample was analyzed via confocal laser scanning microscopy (CLSM; Leica TCS SP8 DMi8; Leica Microsystems, Wetzlar, Germany). For this part of our investigation, the sputtered gold was removed again by washing with water, and the first and clearly visible layer just below the surface was selected for the CLSM tomograms. The confocal micrographs were recorded in dual fluorescence mode with RITC (594 nm) and FITC (488 nm), using an Apochromat 63× oil immersion objective (HC PL APO 63×/1.40 oil CS2; Leica Microsystems) with a resolution of 2048 × 2048 pixels by means of the LASX software (version 3.1.5-16308; Leica Microsystems). The homogeneity of the hybrid layer was visually evaluated. Here, with reference to the distribution of the fluorescence dyes, the confocal micrographs were determined to be homogeneous (well mixed, complete commingling of red and green), heterogeneous (not well mixed, separate red and green areas), or inhomogeneous (not mixed, unevenly distributed).

Both SEM and CLSM images were analyzed by one investigator (J.-S.B.), and this process was repeated (without controlling for group assignment) at 2-week intervals using ImageJ 1.35 S software (National Institutes of Health [NIH], Bethesda, MD, USA) to control previous measurements, and to reduce possible operator errors. The operator had knowledge about the abovementioned group assignment of the eighty tooth quarters, and the captured confocal micrographs were analyzed for both thickness and homogeneity of the hybrid and adhesive layers. A second evaluator, blinded to the various groups to minimize any potential bias (A.M.K.), independently verified all the measurements (ImageJ2, version 2.14.0/1.54f; NIH); in case of any differences based on errors between the repeated measurements, a consent value (without unblinding the image coding) was reached between the investigators. Again, the determined data were collected in an Excel file (Excel 2023; Microsoft) for statistical evaluation.

### Statistical analysis

The present report complies with the SAMPL Guidelines^25^. In the current study, we used a complete block design, with both the patient and the tooth as the experimental unit and without any replicates within a block. By using the permuted block setup, each tooth quarter (mesiobuccal, mesiolingual, distolingual, and distobuccal) was randomly assigned to one treatment strategy (Syntac, Syntac/Icon, Scotchbond MP, Scotchbond MP/Icon; see Fig. 1). Thus, we controlled for any confounding variables by eliminating possible within-tooth heterogeneities and removing interindividual variability from the estimates of the treatment effects (without any carry-across effects), and a well-balanced frequency of the four bonding strategies with the respective tooth quarters was ensured.

Analyses were performed using the SAS 9.4 statistical software (SAS Institute, Cary, NC, USA). Differences between the thicknesses of both the hybrid layer and the adhesive layer were statistically evaluated. For this purpose, the calculated distributions (means ± standard deviation [SD]) of the three-point measurements of all samples were compared; in the case of a given heteroscedasticity, the modified *t* test was used to compare group means, thus decreasing the risk of Type I errors. The visual assessment of homogeneity was determined by variables and was statistically compared (homogeneous = variable a, heterogeneous = variable b, inhomogeneous = variable c). However, with this conventional statistical approach, usually testing the difference between regimens, only conclusions on whether there is enough evidence to reject such an assumption or not would seem possible. Consequently, a failure to reject H_0_ does not necessarily imply that the different treatment strategies (dentine bonding vs. dentine bonding plus resin infiltration) result in the same outcome.

With the current analysis, the equivalence between the various outcome distributions in the study groups was tested. The statistical equivalence concerning the absence of a significant difference with regard to the extent of the thickness of the hybrid layer at the site of action was calculated between the test and the reference groups. Considering that a minimum thickness of 3 to 5 µm would seem sufficient for clinically relevant dentine bond strengths^26^ and that elevated adhesive bond strength is not related to increased hybrid layer thickness (and/or length of resin tags)^27^, a practically important difference would seem mandatory for not acknowledging comparability. Thus, we specified that (bio-)equivalence was achieved if the 90% confidence interval (CI) of the analyzed parameters of the test products was within the lower 90% (–Δ_*L*_) and upper 110% (Δ_*U*_) equivalence bounds of the control group values. To test for equivalence, two one-sided *t* tests (known as the TOST procedure^28^) were applied, meaning that H_0_ referred to inequality. In these combined cases, the overall significance level α was set to 5%, and P values of less than 5% indicated equivalence.

- H_0-1_: The difference between group means should be less than or equal to the lower limit for equivalence (Δ ≤ –Δ_*L*_); and
- H_0-2_: The difference between group means should be greater than or equal to the upper limit for equivalence (Δ ≥ Δ_*U*_).

The thickness of the adhesive layers and the resin tag formation were considered secondary aims; both aspects mainly served for handling control. The adhesive layer thickness was measured and computed, while the dentinal tubules were only visually examined for presence, and no statistical analysis was performed with reference to this outcome.

## Results

The two evaluators’ measurements matched perfectly (except for some tiny micrometer fractions, due to the varying hardware and software used); no data were lost or excluded, and the final dataset was considered representative. With the current setup, no sample case calculation was possible; the used teeth served as the statistical unit, and four specimens each were obtained from among the 20 teeth (n = 80).

**Figure 2:**
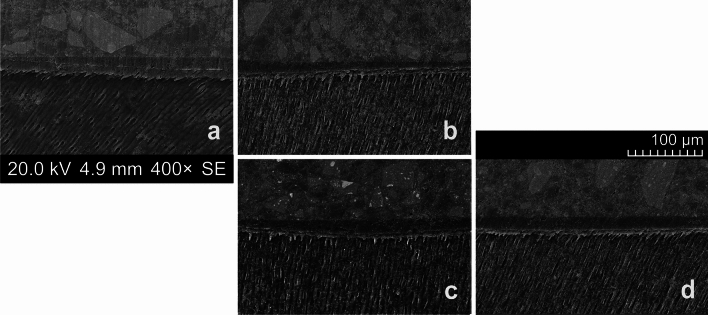
Scanning electron microscopic (SEM) characterization revealing the bonded interfaces under various conditions (magnification factor of 400×). (**a**) Group 1 (control, Syntac Primer/Adhesive plus Heliobond); (**b**) Group 2 (Syntac Primer/Adhesive plus Icon plus Heliobond); (**c**) Group 3 (control, Scotchbond MP Primer plus Scotchbond MP Adhesive); and (**d**) Group 3 (Scotchbond MP Primer plus Icon plus Scotchbond MP Adhesive). With each specimen, the restoration (with filler particles), the adhesive layer, the (partially etched) hybrid layer, and the dentinal tubules (along with resin tag formation) are clearly visible. *SE* secondary electron mode.

**Figure 3:**
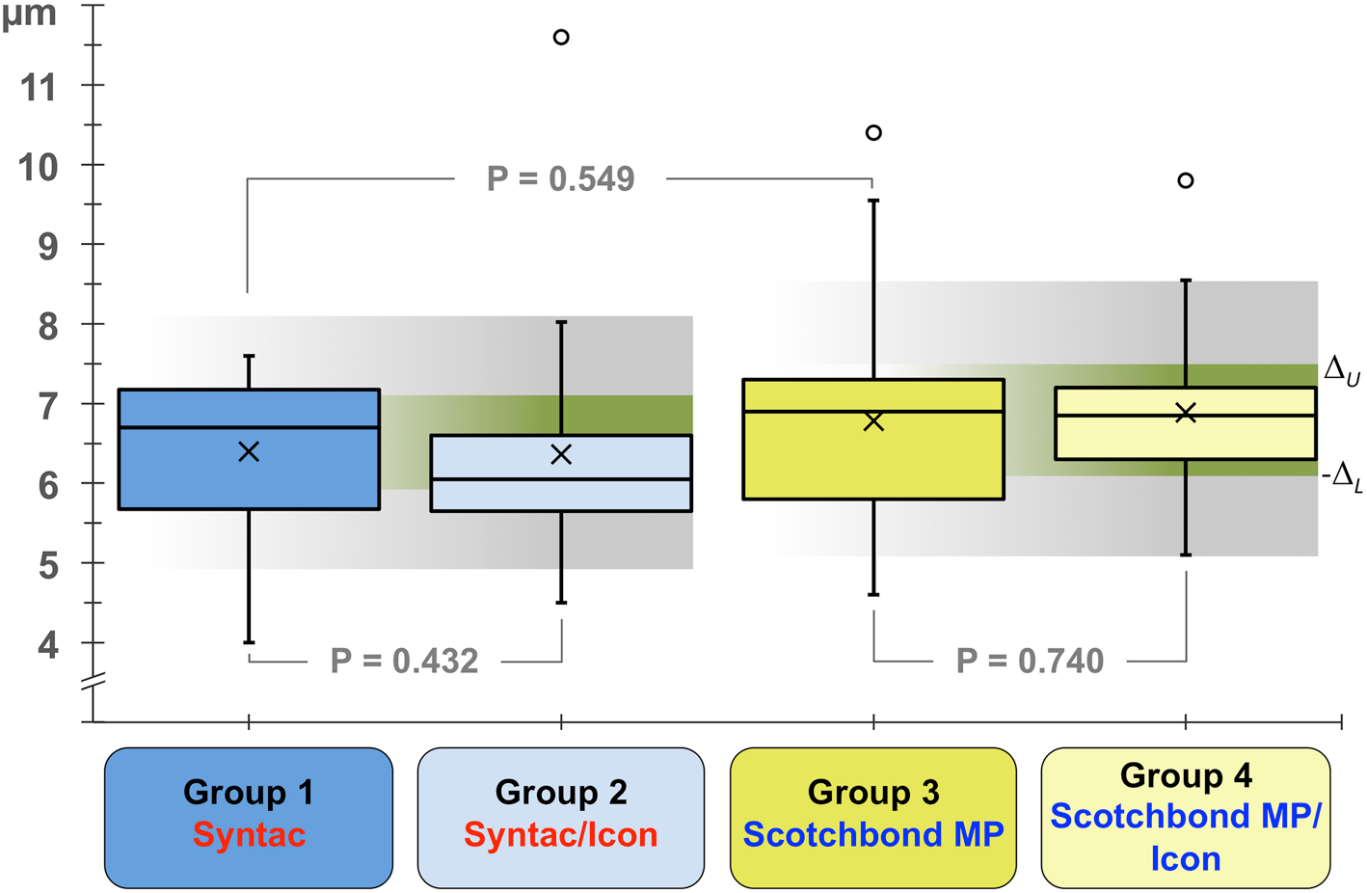
Box-and-whisker plots presenting the outcome referring to scanning electron microscopy (SEM) assessments of the widths of the 4 groups’ resin-dentine interdiffusion zones (hybrid layer thickness). Horizontal lines splitting the boxes indicate medians/second quartiles (${\tilde{\text{x}}}$/Q2), and boxes define upper and lower quartiles, while the ends of the whiskers are set at 1.5× interquartile range above the third quartile (Q3) and at 1.5× interquartile range below the first quartile (Q1). Minimum or maximum values lying outside this range are shown as outliers (**○**), and crosses (×) denote means (${\overline{\text{x}}}$). Given P values indicate non-significant differences after *t* testing between groups, and upper (Δ_*U*_) and lower (− Δ_*L*_) equivalence bounds representing ± 25% and ± 10% of the controls’ means are indicated as transparent grey and green areas, respectively.

Neither the application of maleic acid (Groups 1 and 2) nor the use of itaconic/acrylic acids (Groups 3 and 4) caused noticeable increases or significant differences in the resin-dentine interdiffusion zones (Fig. 2). The mean [SD] thickness of the hybrid layer ranged from 6.4 [1.4] µm (Group 2) to 6.9 [1.2] µm (Group 4), with no significant differences between the controls (Group 1 (6.5 [1.0] µm) and Group 3 (6.8 [1.5] µm); P = 0.549). The hybrid layers after the additional use of Icon appeared uniformly filled, and, when *t* testing for superiority, their widths after priming with Syntac or Scotchbond MP did not differ from those of the controls (Group 1 vs. Group 2, P = 0.432; and Group 3 vs. Group 4, P = 0.740; see Fig. 3).

Testing for equivalence of the various treatment regimens revealed nonsignificant differences when accepting upper and lower equivalence bounds of 10% of the controls’ mean hybrid layer widths (Fig. 3). The TOST procedure resulted in P values less than α, and we could not reveal any significant differences, neither between Group 1 and Group 2 (upper bound Δ_*U*_, P = 0.001; lower bound − Δ_*L*_, P = 0.037) nor between Group 3 and Group 4 (upper bound Δ_*U*_, P = 0.033; lower bound − Δ_*L*_, P = 0.008). Consequently, from a statistical point of view, both H_0–1_ and H_0–2_ were rejected; the observed effects were recumbent within the equivalence margins and close enough to zero to be considered neither superior nor inferior to the respective ranges (thus revealing practical and clinical equivalence).

**Figure 4:**
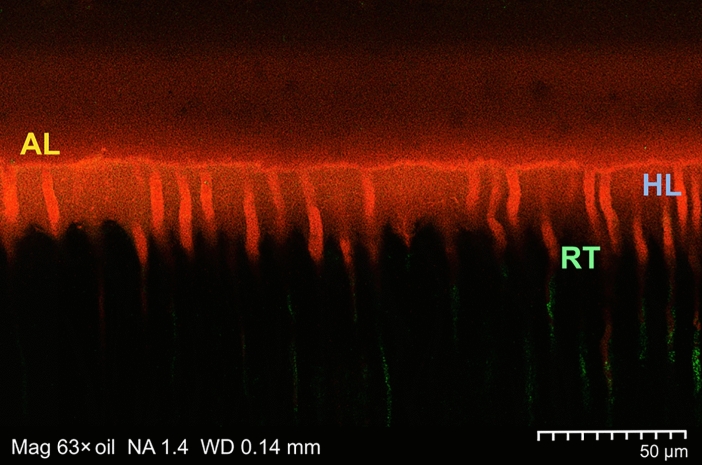
Representative confocal laser scanning microscopy (CLSM) image (using an oil-immersion objective with a magnification factor of 63×) of a Group 2 specimen (Syntac Primer/Adhesive plus Icon plus Heliobond). A well mixed resin-dentine interdiffusion zone (composed of FITC-labeled Syntac Adhesive and RITC-labeled caries infiltrant resin) was clearly visible by means of optical sectioning, thus revealing a homogeneous hybrid layer (HL), along with an adhesive layer (AL). Due to imaging restrictions to the thin z-plane, only resin tags (RT) occluding the dentinal tubules are partially portrayed. *Mag* magnification, *NA* numerical aperture, *WD* free working distance.

**Figure 5:**
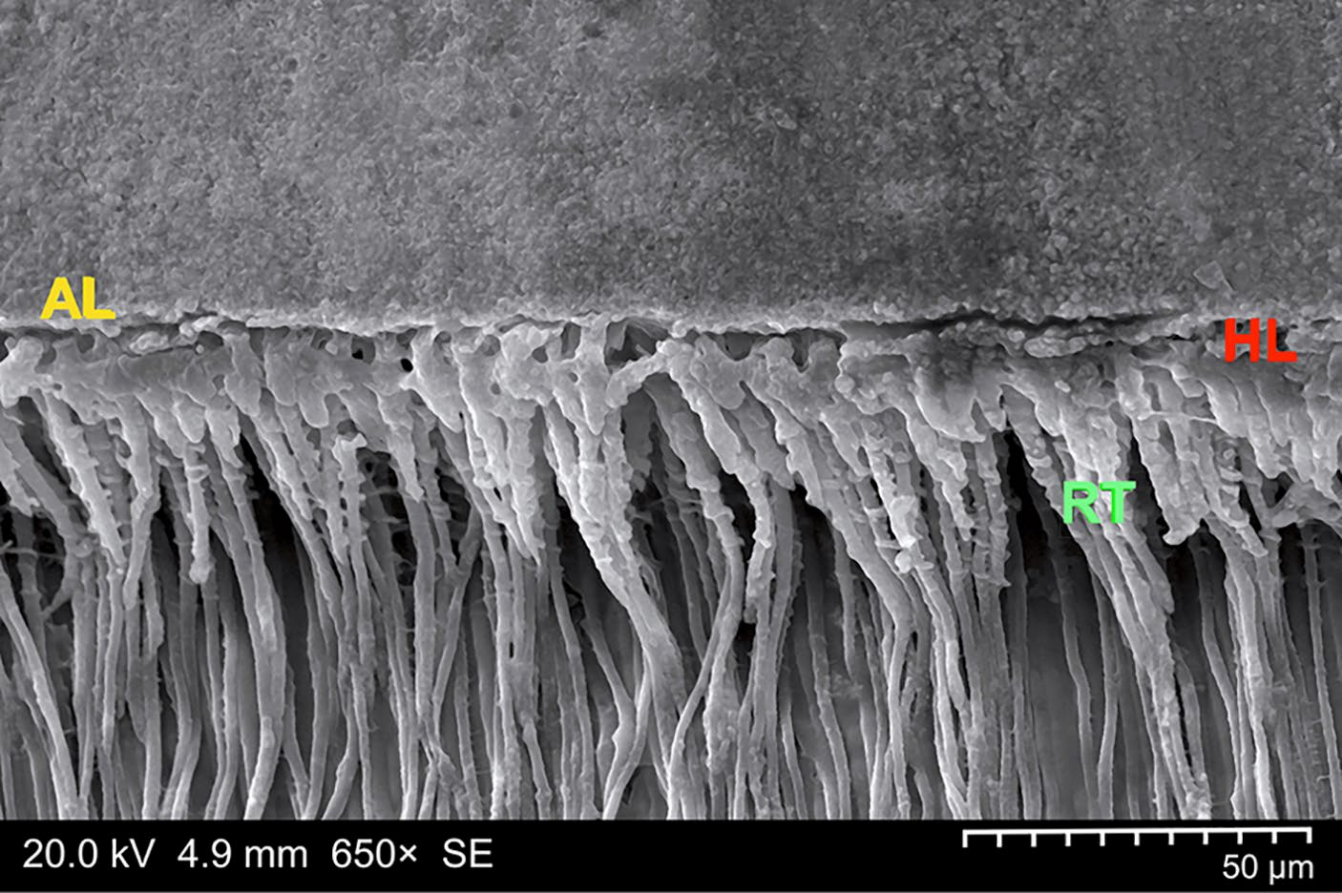
Illustrative scanning electron microscopy (SEM) micrograph, run in secondary electron (SE) imaging mode, of a representative Group 2 specimen (Syntac Primer/Adhesive plus Icon plus Heliobond) observed at a magnification factor of 650×. Note the only partially preserved adhesive–dentine interface, along with the completely dissolved dentine, now unveiling numerous noninterrupted resin tags revealing the excellent penetration capacity. *AL* adhesive layer, *HL* hybrid layer, *RT* resin tags.

With all groups, the hybrid layers as assessed with CLSM were predominantly homogeneous (Fig. 4), with 20/20 homogeneously filled hybrid layers in Groups 1, 3, and 4 (and with 18/20 hybrid layers considered homogeneous and 2/20 specimens rated heterogeneous in Group 2); no specimen was rated as inhomogeneous. SEM observations of the bonded specimens of all experimental groups revealed characteristic reverse cone-shaped resin tags; a representative sample is provided in Fig. 5.

With regard to the mean [SD] widths of the adhesive layers, no significant differences were found between Group 1 (Syntac; 18.1 [13.7] µm) and Group 2 (Syntac/Icon; 20.3 [13.0] µm; P = 0.618) or between Group 3 (Scotchbond MP; 20.1 [14.8] µm) and Group 4 (Scotchbond MP/Icon; 20.0 [10.4] µm; P = 0.978). Again, the controls did not reveal any significant differences (P = 0.549), which could be confirmed when comparing Group 2 and Group 4 (P = 0.184), thus suggesting standardized and comparable application modes.

## Discussion

**Figure 6:**
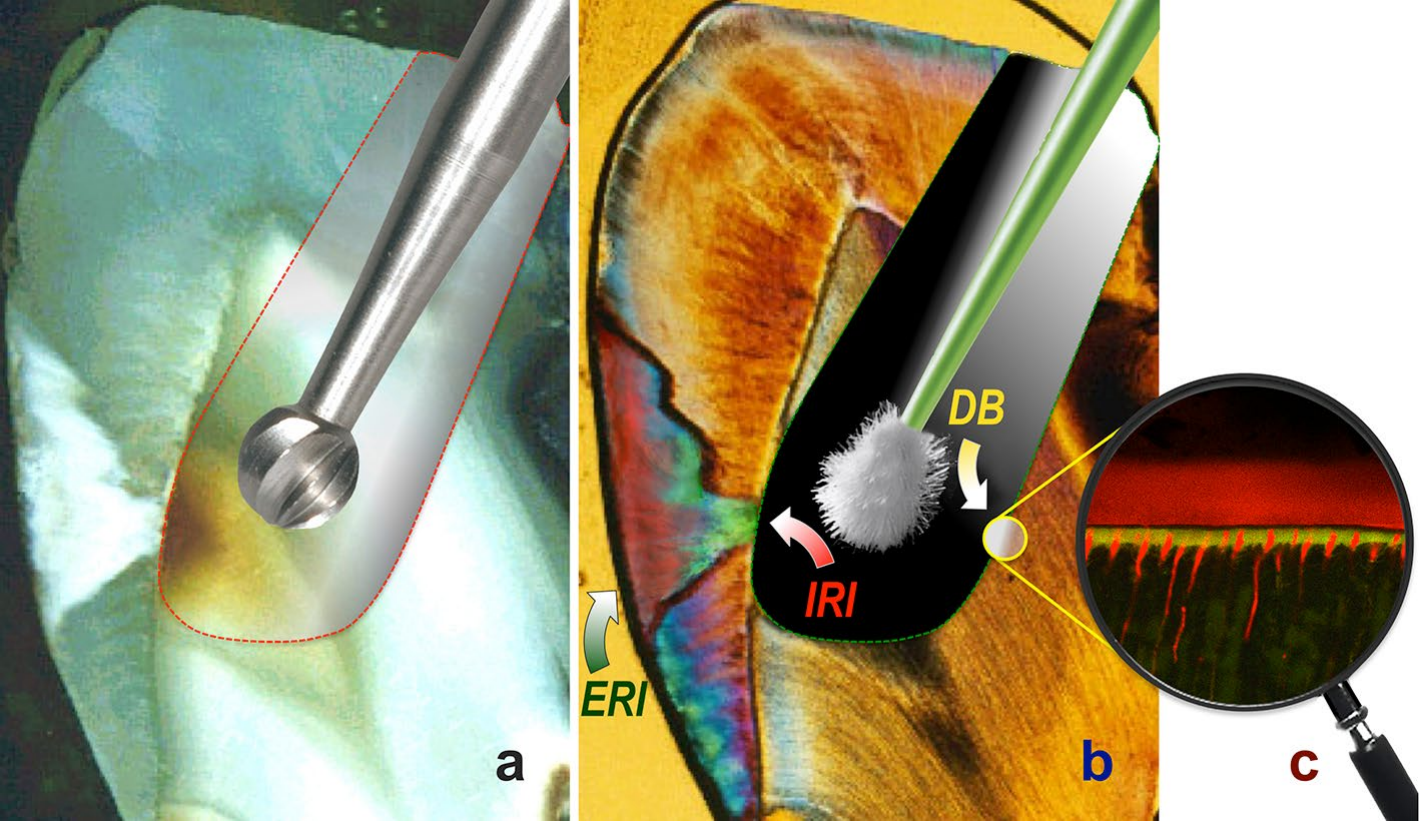
Graphical representation of the present study’s rationale. (**a**) Proximal caries lesion revealing demineralized (but noncavitated) enamel (illustrated by means of a reflected-light microscope); after preparation of a small but adequate occlusal access cavity, the dentine caries will be excavated using a rose-head bur (or, alternatively, with a PolyBur [Komet]). (**b**) Polarized light microscopy image of the same tooth’s thin section (modified from^2^, with permission); after etching and using the ethanol-wet dentine bonding technique, the resin infiltrant will be applied using a microbrush, thus allowing for both internal resin infiltration (*IRI*) of the enamel lesion and dentine bonding (*DB*). (**c**) Confocal laser scanning microscopy image (dual fluorescence mode) revealing the adhesive layer, hybrid layer, and resin tag formation. Treatment will be finished by means of an adhesive Class I (occlusal) composite resin restoration as well as by external resin infiltration (*ERI*), thus stabilizing and protecting the enamel lesion.

When reflecting on the rationale of the current investigation, it would seem noteworthy to re-emphasize that the external resin infiltration technique has been developed to bridge the gap between sound enamel and those enamel lesions, calling for preventive and/or (traditional) restorative treatment^1,2^. According to several independent papers available from the literature, this approach seems to be an accepted treatment option meanwhile, and has been rated as more efficacious than noninvasive (preventive) treatments for halting noncavitated proximal enamel lesions^7,13^. Recently, while recognizing the cumulative evidence, resin infiltration has been recommended for use in active caries by an expert consensus^29^, thus prioritizing the infiltration approach over remineralization attempts^30–32^ and expanding the dogma of minimally invasive dentistry^33^. However, when taking a closer look, it should be clear that at the time of its introduction, none of the preliminarily published papers leading to the development of Icon had focused on lesions extending into dentine^1,2^; indeed, the respective patents dealt only with lesions restricted to enamel. Nonetheless, the manufacturing company unfoundedly extended the spectrum of indications from enamel-restricted lesions (radiographic E1-/E2-lesions) to those spreading across the enamel–dentine border and reaching the outer third of dentine (D1); interestingly enough, this approach has been adopted occasionally, albeit with some degree of restraint^32^. In this vein, it has to be emphasized that proximal radiography frequently underestimates the histological caries depth and extent^34^ (see Fig. 6), and a more accurate detection and diagnosis of proximal lesions should always be made in combination with both clinical and radiographic inspection (along with other complementary methods such as fiberoptic transillumination)^35,36^.

Moreover, it should be clear that radiographically visible proximal dentine caries will accompany higher progression rates, and those enamel–dentine lesions that have been infiltrated apparently share the same fate^10,37^. Notably, after erroneously relying on the manufacturer’s range of indications, subgroup analyses of a 3-year clinical trial on resin infiltration of proximal dentine caries lesions clearly showed success rates of only 64%^38^. These poor results may drop to 50% (with 11/22^39^ lesions having progressed) in permanent teeth or even more with primary teeth (with 4/10^40^ and 9/17^41^ lesions having progressed) after 2 years of observation. In addition, this poor outcome has been corroborated recently, with enamel–dentine lesions rated 59% more likely to present caries progression than enamel-only lesions after external resin infiltration^42^. Thus, to draw a meaningful lesson, external resin infiltration of proximal lesions exceeding into dentine would be comparable to tossing a coin; such poor treatment is unpredictable in its outcome and, hence, is not considered recommendable. Following the manufacturer’s indication range would again be subject to the nondeliberate restorative cycle; the latter includes loss of tooth structure due to the caries process and due to the compelling need to prepare the tooth to receive a restoration, along with occasional failures of the restorations and subsequent demands for rereplacement^43^. Undoubtedly, the current work’s intention is not to stress the difference between cure and treatment, but it should be clear that foreseeable retreatments must be avoided, and this is considered in accordance with the *primum-nil-nocere* percept (‘above all, do not harm’).

To explain the low and unacceptable outcomes described above, the operators’ training and previous experience only fall far short of a satisfactory description (clearly, practical exercises are considered mandatory^42^, and the latter should not be precluded here). In other words, poor handling of the resin infiltration technique should likewise have resulted in higher failure rates with caries lesions restricted to enamel, but this could not be observed in previous studies^38,39,41,42^. Considering the three-dimensional seal of the lesion’s porous volume^1^ (even if the percentage penetration of the infiltrant resin decreases with increasing lesion depth^44^), it would seem expectable that the barrier composed of enamel remnants embedded by the resinous infiltrant should isolate the infiltrated lesion from outside bacteria. However, whether this treatment regimen controls the bacterial burden that has already spread to the dentine^10,45^ has not been determined yet. A speculative conclusion by analogy (from studies having investigated the sealing of carious fissures) would suggest a drastic reduction in the mean total viable bacterial count^46^, thus concomitantly leading to clinical and radiographic slowing down or even arrest of lesions extending into dentine^47–49^. Unfortunately, as far as proximal lesions are concerned, reliable data on this aspect are currently unavailable.

With these thoughts in mind, particular attention should be given to a further aspect considered underreported in the past. Recently, it has been shown that the incidence of enamel craze lines (with gradual propagation to microcracks over time) in proximal lesion areas of teeth increases in relation to the extent of demineralization^50^. This could explain why propagated chipping and other (micro)defect rates corresponded to advanced lesion depths^51^. Moreover, enamel lamellae have been revealed to provide permeable pathways, allowing microorganisms to cause caries access to the enamel–dentine junction^52^. Together with the subsequent and extensive microbial contamination of those (micro)cracks^53,54^ this may act as a predisposing factor for proximal caries progression^50^. Interestingly (and even beneficial), resin infiltration of carious fissures (as has been shown previously^55^) will dramatically augment crack initiation load values, at least when evaluated by means of 3D computational models^56^, and this strengthening effect would seem transferable to proximal lesions, particularly those that have been infiltrated both internally and externally. Indeed, a recent study revealed the efficacy of resin infiltration for the treatment of clinically symptomatic enamel infractions^57^, thus suggesting the stabilization of those defects and craze lines, along with both the inhibition of microbial colonization and the prevention of staining.

The radiographic lesion depth correlates with the level of bacterial infection^45^ (and this equally refers both to noncavitated^58^ and to nonactive^59^ lesions), along with an accumulation of proteins (‘subsurface pellicle’)^60^, microbial byproducts^61^, lipids, polysaccharides, and/or other salivary or food constituents^62^, as well as water and air^63^, all of which have an unclear total impact^64^ but probably impede complete remineralization^65,66^. Consequently, these comparably high water and organic volumes^67,68^ would call either for a conventional Class II cavity or for a reasonable and controlled alternative. The latter has been proposed recently, and the combination of internal and external infiltration of the enamel lesion (including the deproteinization of the latter^5,67^ to increase the comparably low proportion of the pore volume considered available for resin infiltration^63,69^) would seem to be a complimentary reinforcement of the internal tunnel approach^14,15^. Early studies have revealed poor success rates of traditional (complete) tunnel preparation, which can be attributed to the comparably low adhesive forces of the glass ionomer (and amalgam) restorations^70^ frequently used with this technique, as well as to the inherent sensitivity of this technique and the operator’s corresponding experience^71–74^. However, more recent investigations focusing on adhesive dentistry have elucidated various benefits of using this treatment option when combined with adhesively luted composite resins^70,75–82^. These advantages may be summarized as adhesive strengthening of the remaining tooth structure, and this will be complemented by an acceptable marginal seal quality as well as by reduced wear rates of the comparably small volume and small occlusal contact area of the composite resin restoration^83^.

Recently, it has been corroborated by means of a modeling study clearly exemplifying that preserving the integrity and anatomy of the proximal marginal ridge is considered essential for minimizing cuspal flexure and maintaining the mechanical strength of the tooth^56^. Consequently, tunnel preparation provides a greater mechanical advantage than conventional Class II or box-only preparation methods^84^, thus protecting the restored tooth from fracture. Combining the internal tunnel approach with two-sided resin infiltration of the enamel lesion would further improve tooth strength, and still would constitute a minimally invasive method for treating proximal caries, in particular if compared to restoring (saucer-shaped) Class II cavities. Undoubtedly, the biomechanical performance of the restored tooth will improve when this method is used^85^. Additionally, this refined treatment concept^14,15^ ensures the removal of dentine caries (through the internal tunnel approach) while preserving the proximal enamel wall (including the sound proximal contact area and the marginal ridge). Moreover, the porous volume of the enamel caries will be occluded by double-sided resin infiltration, thus adhesively strengthening the lesion area and maintaining the interproximal contour and surface (including this region’s physiology); these areas are now considered to be able to resist abrasion or attrition and to prevent cavitation (compare Fig. 6).

In the present study, we have used Syntac (containing tri-ethylene glycol dimethacrylate [TEGDMA], both with Primer and with Heliobond) as a dentine bonding agent, and this multibottle system would seem compatible with Icon. Glutaraldehyde (as an ingredient of Syntac Adhesive) is a well-known cross-linking (fixating) agent with a high affinity for the primary amine groups of the collagen that reduces degradation of the latter, thus preserving its properties^86^. In contrast, the equally utilized Scotchbond MP is a two-bottle adhesive containing 2-hydroxyethyl methacrylate (HEMA), a monomer known to improve the impregnation and envelopment of collagenous components of demineralized dentine^87^. Owing to its pendant hydroxyl groups, HEMA is entirely miscible with both water and ethanol; to some degree, the monomers will crosslink to poly(2-hydroxyethyl methacrylate [pHEMA]), which is considered insoluble in common solvents and nondegradable^88^. As with TEGDMA (which is a typical cross-linking additive), the inclusion of HEMA in the polymer matrix of composite resins has a positive effect on the properties of the latter, thus improving the hardness, diametral tensile strength, three-point bending strength, flexural modulus, and shrinkage stress^89^. With the current investigation, homogenous mixing of the various monomers along the interfaces could be revealed for the different conditions studied by means of CLSM (see Fig. 4), thus confirming previous outcomes regarding both homogeneity and continuity of the hybrid layers^90^.

When combining external/internal infiltration with the internal tunnel approach, after excavating the dentine caries via the occlusal access cavity, a smear layer will occlude the dentine site of the enamel lesion. To ensure high internal infiltrability of the enamel lesion, this smear layer must be removed, which can be achieved by means of using a phosphoric acid gel^14,15^. When deploying the latter in a comparably small cavity, however, no selective etching will be possible, and this would suggest an etch-and-rinse (total-etch) regimen^91^ to additionally remove the dentinal smear layer (notably, residual remnants of the latter might have inhibitory effects on polymerization networks), at the same time exposing both collagen fibers and dentinal tubules. With the applied etching time (60 s, followed by the compulsory rinsing with water), the dentine surface was demineralized to a depth of approximately 6 µm in the current study, and this corresponded well to values reported in the literature^26,92^. This demineralized dentine surface constitutes the fundamental prerequisite for dentine hybridization; here, an interdiffusion zone called the hybrid layer (which is composed of residual hydroxyapatite, collagen, resin monomers, and solvents) is created. This hybrid layer forms the basis of micromechanical retention of the composite restoration to be adhesively entrenched to the dentine (Fig. 2). When focusing on bonding performance, it should be underscored that the latter is influenced by the technique (but is less dependent on bonding materials used); thus phosphoric acid etching is recommended in favor of any mild priming agent^93^ as a routine treatment with established efficacy, as has been confirmed with the present study. Nonetheless, while the quality of the hybrid layer influences adhesion to dentine (depending on the adhesive used), the length of the resin tags (compare Fig. 5) should not have any impact on the bond strength.

However, with the current approach of internal (enamel) infiltration in mind, it must explicitly emphasized that, after total etching and prior to application of the resin infiltrant, a thorough air-drying of the enamel lesion is mandatory, and this will undoubtedly result in a collapse of the dentine collagen network previously exposed by means of the etch-and-rinse procedure. When remembering that the application of alcohol to remove the water from the porous internal enamel lesion site would seem advisable, this directly leads to the ethanol-wet dentine bonding technique^94^. With its higher vapor pressure (compared to water), ethanol (Icon Dry) facilitates water evaporation (including the water applied with Syntac or Scotchbond MP Primer), at the same time allowing for a reduced re-expansion of the collagen fibrillar network if previously dried (or, in other words, a slightly greater shrinkage, if compared to water-wet dentine). This, in turn, will result in an exiguously decreased hybrid layer thickness characterized by high quality^95^. Moreover, ethanol dehydrates water-trapping proteoglycans usually filling the interfibrillar spaces (at the same time causing only mild pulpal damage, thus being comparable to water-wet bonding^96^); this results in the shrinkage of those hydrophilic organics (along with reduced diameters of the collagen fibrils) and leads to widened spaces between the latter, now serving as infiltration pathways for the dentine bonding monomers. Subsequently, infiltration of hydrophilic and/or hydrophobic dimethacrylates into the ethanol-imbued hybridized dentine is improved^97^ but is not influenced by the solvent content of the bonding agent^98^. As has been demonstrated with the present findings (see Figs. 2, 4, and 5), this simplified ethanol-wet bonding would seem suitable with the etch-and-rinse adhesives used with the current setup^94^.

With the ethanol-wet dentine bonding technique, the resulting hybrid layer is characterized by a reduced number of water voids, a higher resin-collagen ratio, and increased resin encapsulation of the demineralized collagen network^99^; this layer should include exposed/acid-activated endogenous matrix metalloproteinases (MMPs, requiring water to function) and cysteine cathepsins, both of which are considered responsible for collagenolysis. It should be emphasized that a complete impregnation of the open spaces of the collagen network with adhesive resin would seem crucial for preventing enzymatic degradation, thus safeguarding the long-term integrity of the collagen fibers, and, consequently, the long-term stability of the hybrid layer. Of equal relevance and importance, avoiding possible water voids will reduce incomplete infiltration rates, insufficient monomer conversion, and hydrolytic degradation of adhesive resins that have encapsulated and saturated the collagen matrix. While any trapping of water within the adhesive will result in incomplete polymerization, hydrolysis refers to the reaction of water with the ester bonds of polymers or macromolecular monomers (such as TEGDMA), and this process leads to a breakdown of the polymeric chains^86^. The elution of (broken) resin monomers might be facilitated by water voids, which will be enhanced by future water ingress through poorly polymerized (or underfilled) areas and hydrophilic domains^100^. The effects described above could explain infiltrated enamel white spot lesions showing discolorations after storage in staining solutions^101^; interestingly, bleachability of such infiltrated and stained areas has been shown^102^, and this again would substantiate rough or incompletely infiltrated areas. Careful and complete application (including polishing, thus removing any oxygen inhibition layers), in contrast, will result in infiltrated white spots that have been considered aesthetically stable in clinical trials over a period of more than 4 years^103,104^, thus suggesting that careful application and complete polymerization will lead to a complete infiltration, both with enamel and with dentine.

In addition to the abovementioned ethanol-wet dentine bonding technique, further strategies aimed at improving the quality of the hybrid layer include careful but prolonged blowing and drying (thus ensuring water and ethanol evaporation) and increased active application (rubbing) time of the adhesives (thus increasing penetration of the demineralized collagen network), and the latter has been shown to sufficiently compensate for the effects of prolonged etching periods^105^. Furthermore, extending the curing time (thus safeguarding polymerization) and applying a hydrophobic resinous coating (thus impeding re-entry of water)^106,107^ will ensure the desired quality of the hybrid layer. It should be emphasized that all these modifications deviate from the widely applied procedures but clearly should provide improved bond degradation prevention; this improvement is based on the increased quality of the hybrid layer (but not on the multiple adhesive applications leading to a simple increase in thickness), and this should ensure both immediate and aged bond strengths. With the current setup, all those aspects have been taken into account; in particular, we have included a thoroughly extended application time of the resinous infiltrant (Icon) which was massaged into demineralized and ethanol-wet dentine (concurrently allowing infiltration of the enamel lesion from the inner cavity site, as has been shown previously^14,15^). Ethanol has been proven to be a better solvent for higher-molecular-weight monomers than water; the application approach described in the present study (revealing comparable widths of the adhesive layers and confirming a uniform handling of the various bonding agents) aims to reduce water droplets/pores within the polymer, thus impeding future fluid flow into the adhesive-dentine interface^108^. Moreover, the present ethanol-based concept contributes to improved dentine wettability^109^, reduced collagen degradation, decreased monomer hydrolysis^110^, and improved dentine bond durability, thus ensuring generally accepted antidegradation strategies^111^.

Here, we introduce a new methodology for a novel bonding strategy, aiming to ensure both resin infiltration of the inner enamel lesion and adhesive dentine bonding of the restoration. In this way, the latter should overcome previous handling shortcomings (such as hindering the adhesion of the restoration to the bonding surface or overdrying the dentine after phosphoric acid etching). However, this concept clearly deviates from the instructions originally advocated by the respective manufacturers; notwithstanding, when aiming at an optimal result and when considering the longest possible durability of the resin restoration as the ultimate objective, strict adherence to this approach would seem recommendable. When reflecting on established restoration techniques, even minor deviations from established bonding strategies should be avoided (due to increased failure rates)^112^. However, the proposed concept refers to a bonding strategy combining both, carious enamel and demineralized dentine infiltration. Consequently, oversimplification trends as observed with recent dentine bonding techniques^113^ would not seem to be an expedient approach when keeping in mind the twofold objective presented here to establish a durable bond. With the current setup, albeit more laborious than other bonding techniques, a sufficiently long (3 min) and active (scrubbing) application of the resinous infiltrant was established (including an adaptation of the Heliobond and Scotchbond MP Adhesive application times). Indeed, this double-layer application approach leads to increased resin saturation of collagen by monomers, resulting in blockage of the space between the exposed collagen fibrils. The final and prolonged air drying (20 s) should have resulted in an increased evaporation of the solvents^22^ possibly hindering the formation of cross-linked polymers owing to a physical phase separation (into hydrophobic and hydrophilic-rich phases, in particular in the presence of water)^114^. This should lead to improved inward diffusion of the monomers to increase impregnation; indeed, with our current SEM and CLSM analyses (both considered complementary and synergistic methods^115^, compare Figs. 4 and 5), we did not observe any remaining blanks.

With the development of the resin infiltration technique^116–119^ one aim was to provide a material considered compatible with current materials used in adhesive dentistry, and this has been confirmed previously^2,55^; with the current experiments, compatibility of the TEGDMA and HEMA components used with the various groups could be revealed. From a physico-chemical point of view, Icon is a low-viscosity, unfilled light-curable resin characterized by a high penetration coefficient (> 200 cm/s) based on fully aliphatic TEGDMA (a widely used diluent in many resins and adhesives), with ethanol as a solvent and with camphorquinone (CQ) as a photoinitiator; the absence of fillers assures the low viscosity of Icon. TEGDMA is a very short monomer, and with its small distance between the double bonds, the crosslink density increases^120^. With elevated TEGDMA concentrations (as in the case of Icon use), the high degree of conversion results in a homopolymer, characterized by a compact network^121^, while the possibility of chain reorganization is greatly reduced; chemical crosslink density, physical crosslinking, and degree of conversion will positively affect impact resistance^120^. The quality of various other parameters (such as solubility, water sorption, depth of cure, efficient copolymerization between the adhesives and the primed dentine, bond strength, and flexural strength of this densely packed adhesive^122,123^) would seem to be an acceptable compromise (if not considered a perfect one) to achieve both sufficient dentine bond quality and improved adhesive strength. Consequently, the presented approach (using the sequences provided with Table 2) would seem suitable for both hybrid layer formation and internal infiltration of carious enamel.

However, hydrophilic monomers such as TEGDMA (as well as HEMA) are considered leachable from bonding resins^124,125^, and this refers to both nonpolymerized remnants and degraded (or derivative) components. Consequently, when appraising the bonding strategy presented with the current concept, aspects of cytotoxicity must be elucidated. Although TEGDMA has been rated less toxic than other methacrylates^126,127^ and some studies have shown that neither TEGDMA nor HEMA can alter the normal differentiation and mineralization process of pulp cells^128^, this aspect has recently become a matter of debate, since even low TEGDMA concentrations might already have a pulpotoxic impact^129,130^. This also refers to odontoblast cell dysfunction shown in response to TEGDMA and HEMA monomers^131^. Following direct contact with pulpal stem cells for 48 h, nonpolymerised Icon dramatically reduced cell viability (up to 98.9%), whereas light-cured Icon revealed an only modest cytotoxicity (down to 10%)^132^. Diffusion into the pulp (and thus toxicity) will strongly depend on the remaining dentine thickness and its respective histological changes (dead tracts and sclerotic dentine, as shown in Fig. 6b). Other influential factors include the positive pressure of the dentinal fluid via the dentinal tubules, the degree of conversion and crosslinking (polymer network), the availability of solvents, any possible enzymatic activity, and the monomer concentration^131,133^. Moreover, glutaraldehyde and serum albumin in dentinal fluid can precipitate and block the dentinal tubules^134^. Consequently, excessive resin penetration and tag formation as revealed in Fig. 5 are not considered probable under clinical conditions; since the reported effects have been elucidated via laboratory studies, further in vivo investigations to gain a valid and complete assessment of the toxicity risk would seem mandatory^135^. Nevertheless, careful and complete light curing^24,121^ to increase the degree of conversion must be secured with the present approach (as with all dentine bonding agents^126,132,136^), and this not merely (but above all) should be emphasized with deep cavities^23^.

Taken together, the equivalence of the tested materials and the respective combinations has been demonstrated with the current outcome; neither the hybrid layer thickness nor the homogeneity of the hybrid layers differed significantly between the four tested groups. Moreover, the differences between the means were not outside the equivalence intervals, and it would seem clear that even a 25% equivalence corridor (indicated as transparent/gray areas, see Fig. 3) would have led to sufficiently thick hybrid layers (of 3–5 µm^26^); with the present outcome, we could reveal an even smaller Δ value of ± 10%. For the sake of completeness (although aware of the direct relationship between P values and this kind of observed power statistic), it should be noted that the post hoc power values referring to the 10% level of tolerated deviation for the equivalence tests ranged from 0.983 (− Δ_*L*_, Group 1 vs. 2) to 0.993 (Δ_*U*_, Group 3 vs. 4). This was considered sufficiently high to reduce the chance of Type I and Type II errors (please remember that equivalence testing is an adaptation of traditional significance tests and that the error types are interchanged) and to draw any reliable conclusions. From a statistical point of view, the number of repetitions was considered adequate, and the sample can be considered representative; both the replicability and the reproducibility suggest generalizability and a sufficiently high level of external validity. However, prior to possible clinical translation, the authors acknowledge the limitations of the present ex vivo work, as the complex dynamics of the clinical situation could not be completely mimicked. Accordingly, thermal cycling, artificial aging, and bond strength testing (along with reduced application times) would be important aspects to consider in future trials.

Nonetheless, the present results underline that it would seem possible to generate a compact and homogeneous hybrid layer, and the latter would suggest that, owing to the chosen setup using the human (split-)tooth as the statistical unit and mitigating any possible bias, a sound and valid proof of concept has been provided with the current experiments. The technique introduced here will postpone (or even prevent) restorative intervention in the case of proximal dentine caries by combining the noninvasive (internal/external) infiltration of enamel caries with an adhesively luted conservative occlusal restoration. Improved adhesive tooth strength of tunnel restorations has been confirmed many times, and this refers to sufficient reinforcement of resistance to normal masticatory function^76,79,81^ as well as to total clinical success^70,75,80^. Combining the internal tunnel approach with double-sided infiltration^14,15^ will lead to additional adhesive re-enforcement, and to an increased mechanical strength of the demineralized enamel (with a high surface hardness and remaining stable following thermocycling and water sorption challenges)^6,137^. This novel tunnel technique approach also requires significantly less time than the traditional concept of preparing proximal boxes (with all its associated disadvantages). Consequently, this new treatment concept (enabling a much more durable and less loaded occlusal restoration) bears significant implications for both dental research and clinical practice, offers a new perspective on the therapeutic decision on proximal carious lesions exceeding the dentinal border, and should be a valid alternative to conventional Class II cavities, particularly since marginal ridge preservation is ensured and because the interproximal contour can be maintained with this regimen.

## Conclusion

According to the outcome of the present ex vivo investigation, Icon can be successfully integrated into an ethanol-wet dentine bonding strategy following an etch-and-rinse approach using multibottle adhesives. With increased solvent evaporation, the application of multiple layers (including a hydrophobic coating), and the extended polymerization time, means and methods as presented will lead to a fully fledged hybrid layer. Along with the ultraconservative internal tunnel technique (occlusal Class I cavity only), the concept presented in the current study should complement the spectrum of minimally invasive dentistry, thus postponing or even preventing Class II restorative interventions by noninvasive internal and external infiltration of the proximal enamel lesion.

## Data Availability

Prior to the start of the present investigation, the study protocol has been preregistered. Both the latter and the raw data providing the basis for the findings of this study are available at protocols.io (https://www.protocols.io/blind/E5754DAFD67D11EEB9800A58A9FEAC02) for non-commercial purposes.
